# Translating screen-media guidance into caregiver practices: moving from digital silos to shared spaces for young children

**DOI:** 10.3389/fsoc.2026.1923050

**Published:** 2026-09-25

**Authors:** Jahau Lalnunmawii, Sreeja P. Gangadharan

**Affiliations:** Department of Psychology, CHRIST (Deemed to be University), Bengaluru, India

**Keywords:** childcare arrangements, family sociology, parenting practices, screen time guidelines, spatial constraints

## Abstract

Screen time guidelines for preschoolers have heavily relied on duration controls. Recently, guidelines have evolved to focus on the content and context of use; however, digital health impacts are still widely measured against guideline targets that apply a one-size-fits-all assumption. Despite these recommendations, a global meta-analysis shows that only 35.6% of children under age five adhere to these guidelines. This low compliance is driven by contemporary lifestyle demands, busy parental schedules, and constrained urban spaces. Compounded by socioeconomic disparities and a lack of practical ideas for alternative activities, screens have become a default domestic childcare arrangement, escalating passive, isolated consumption. This paper introduces a three-layered ecological framework that bridges the gap between the targets of institutional guidelines and the diverse ground realities of managing digital engagement across different socioeconomic statuses. It lays out a practical blueprint to operationalize these objectives through a protocol that integrates screen awareness, meaningful alternatives, active engagement, responsive caregiving, and traditional games. This framework provides actionable guidance to help families transition from digital silos to interactive, shared spaces, aiming to alleviate parental stress and support the parent–child relationship.

## 1 Introduction

Children naturally seek engagement and interaction with their immediate surroundings. Sensory stimulation, environmental exploration, and play are universal phenomena essential to foundational development, acting as the primary engines for early childhood learning (Parker et al., 2022; Çankaya et al., 2023). During this formative phase, children rapidly build cognitive functions, language acquisition, emotional regulation, and motor skills through active physical and social interactions (Gallardo-Rodríguez et al., 2023). Rapid urbanization, changing family dynamics, and contemporary lifestyle demands heavily restrict active play choices within the home environment (Mygind et al., 2026; Loebach et al., 2021). The COVID-19 pandemic lockdowns substantiate the challenges faced by parents as families had to stay in confinement, creating a barrier for children to naturally engage with their physical environment (Kharel et al., 2022). The lockdown led to an escalation in prolonged screen time exposure which induced detrimental effects on cognitive function, language acquisition, executive function, and social–emotional growth (Sticca et al., 2025). These types of constraints compel parents to rely on digital devices as babysitting tools, leading young children into cycles of passive media consumption (Nikken, 2019; Panjeti-Madan and Ranganathan, 2023).

Passive screen time consumption is a widespread public health concern as many families struggle to meet screen time guidelines. Globally, a meta-analysis shows only 35.6% of children under age 5 meet the screen time guidelines recommended by the World Health Organization (WHO) (McArthur et al., 2022; Willumsen and Bull, 2019). In Asian countries, 33.7% of young children follow screen time guidelines in Japan and 31% in Bangladesh (Kim et al., 2020; Hossain et al., 2023). In India, the challenges appear to be amplified with 60 to 90% of preschoolers exceeding the recommended screen time (Bhutani et al., 2025; John et al., 2021). However, there are also wide regional variations across Asia with 88.2% adherence in Beijing and 70.2% in Singapore, respectively (Guan et al., 2020; Chen et al., 2019).

While these cities show high adherence through targeted approaches, institutions like the World Health Organization (WHO) (Willumsen and Bull, 2019) and other health organizations continue to maintain these duration-based guidelines (Nuvoli et al., 2025). The WHO acknowledges that there is poor evidence linking screen time duration to physical health problems (World Health Organization, 2019). Recognizing the importance of the content and context of screen use among preschoolers, the Indian Academy of Pediatrics (IAP), American Academy of Pediatrics (AAP) and Canadian Pediatrics Society (CPS) have also evolved from traditional duration-based recommendations to prescribing digital hygiene (Gupta et al., 2022; Hill et al., 2016; Ponti et al., 2017). However, these guidelines appear to provide limited operationalization and contextual implementation, and may not fully account for the ground realities and challenges of modern lifestyles. Parents generally understand the importance of limiting screen time, but struggle with a lack of ideas for alternative activities. Due to these daily lifestyle constraints, screen time continues to be one of the most convenient tools to engage children (Varadarajan et al., 2026), highlighting the mismatch between guideline targets and domestic practice.

To address these challenges, this paper introduces a three-layered ecological as a conceptual framework. Existing socio-ecological frameworks of screen use consider the micro-level environment as relevant but present it as one layer within a broader framework spanning individual, caregiver, and wider socio-environmental levels, without specifying the detailed actions caregivers should take (Morawska et al., 2023; Ragasa et al., 2024; Munzer et al., 2026). This framework positions the caregiver and the micro-level environment as one integrated focus for change, providing operationalized caregiver-executable actions through the SMART protocol. It is intended to provide accessible and practical applicability by contextualizing existing guidelines into actionable recommendations that primary healthcare providers can deliver through pathways suited to the individual requirements of each family, with the goal of helping transform digital silos into shared spaces.

## 2 Institutional screen time guidelines and their execution limitations

Major health institutions like the IAP (Gupta et al., 2022), AAP (Hill et al., 2016), and CPS (Ponti et al., 2017) have implemented screen time guidelines for young children due to the rising concerns on excessive digital screen exposure. These institutional recommendations have successfully shifted focus from simple duration-based restrictions to conceptual approaches and guidelines focusing on content and context. The AAP was the first institution to acknowledge the importance of the content and context of screen use (Hill et al., 2016). This was followed by the CPS, which introduced the 4Ms approach in 2017: Minimizing screen time, Mitigating the risk associated with screen time, Mindfully using screens, and Modeling healthy screen habits (Ponti et al., 2017). In 2022, the IAP also implemented media guidelines encompassing screen limits and digital hygiene rules such as selecting quality content, active co-viewing, and not replacing essential activities with screen time (Gupta et al., 2022). In 2024, the AAP introduced the 5Cs approach, which shifted the duration focus to the content and context of use: Child (Consider the child’s unique temperament, age, and behavioral traits), Content (Select age-appropriate content, appropriate modeling, and avoid violence), Calm (Offer alternatives to regulate emotions without using screens), Crowding Out (Ensure screens do not displace family time, sleep, or physical play), and Communication (Educate children on media literacy) (Williams and Radesky, 2024). Recently, the AAP 2026 policy statement expanded these guidelines by formally introducing a four-layered socioecological framework comprised of the child, the caregivers, the digital ecosystem, and wider social systems (Munzer et al., 2026). This represents the most current institutional attempt to widen the frame beyond individual child behavior and screen duration alone. This is a positive evolution and improvement on paper, but provides largely face-value information on child-appropriate content and the context of digital screen engagement, leaving an operational gap on how parents can curate a healthy and active screen time, neglecting the accessibility of resources and selection of alternative activities to replace screens.

The behavioral requirements, such as Minimizing (CPS), Modeling (CPS), Mindfully using (CPS), and Crowding Out (AAP), demand changes in daily habits without accounting for a family’s daily lifestyle and availability of materials (Nuvoli et al., 2025; Hill et al., 2016; Ponti et al., 2017). These guidelines provide limited baseline explanation for selecting structural media quality like visual and auditory pacing for Child (AAP), Content (AAP), or Mitigating (CPS) components given in these approaches (Hill et al., 2016; Ponti et al., 2017). They prescribe minimal instructions or alternative tools to satisfy the Calm (AAP) and Communication (AAP) directives (Hill et al., 2016; Ponti et al., 2017). For example, a mere suggestion on co-viewing can be taken as simply watching screens together without clear instructions on how to interact, leaving busy parents unable to actively engage with their children’s screen time. This limitation persists even within the AAP 2026 socioecological framework, where naming caregivers as a critical layer of the framework does not, on its own, specify the operational steps that caregivers should take during screen use. The existing guidelines prescribe what targets to reach, where caregivers are directed toward a goal without defined step-by-step instructions, methods, or activities to achieve these targets, within their unique domestic environments. The current guidelines may ambiguously direct screen use, potentially disrupting the verbal and sensory stimulation critical for supporting early cognitive, language, and physical development.

These policies present uniform guidelines without catering to individual developmental needs, presenting an operational challenge for families with constrained living spaces and limited resources. Families living in urban high-density apartments lack safe outdoor space due to increased road traffic, and are unable to create the shared physical space expected by the guidelines (Draper et al., 2022). On the other hand, families living in rural areas may have open space but face a scarcity of tangible play materials and have different daily domestic routines (Varadarajan et al., 2026). Due to differences in domestic environments, the universal guidelines face significant barriers to realistic execution as they do not provide clear directions for families living in these conditions (Shah et al., 2019).

The theoretical guidelines provide conceptual targets without considering the diverse physical realities for implementation. For instance, guidelines use terms such as “Mindfully Using” or “Crowding Out” without providing busy caregivers with the necessary instructions on how to execute these goals, limiting the practical applicability of the guidelines for supporting healthy child development. Qualitative evidence indicates that this gap between guideline demands and daily domestic realities is associated with confusion, limited access to clear information, and parenting stress among families (Ly et al., 2026), suggesting that managing screen time may reflect a structural challenge rooted in the absence of actionable micro-level pathways to implement existing guidelines, rather than a simple parenting concern.

## 3 The bioecological displacement of active unstructured play by digital screen consumption

The disparity between the guideline expectations and the ground realities of families can be understood through domestic struggles and environmental constraints. The Bioecological model shows that human development is influenced by the reciprocal interactions with the immediate surroundings where the type of engagement depends upon the characteristics of the individual, which are co-constructed through continuous interaction with the environment (Bronfenbrenner and Morris, 2006). Analogue play becomes an integral part of the developmental system as it enables physical exploration, active play, and interactions with the environment that are essential for language, cognitive, sensory, motor, and socio-emotional development (Istenič Starčič et al., 2023). Shared space and social interactions are a part of children’s daily lives, and they spontaneously initiate play within their immediate environments. With rapid technological advancement, urbanization, social constraints, and contemporary lifestyles, it becomes challenging for parents to find indoor alternative activities, compelling them to introduce digital screens (Keya and Choudhury, 2025). Due to this convenience and accessibility, parents use screen time as an instrument to entertain and occupy their children, replacing their surroundings and interactions with a virtual environment, marginalizing traditional and unstructured play.

The digital screen environment can inhibit children from experiencing their natural surroundings, creating different exposures between analogue play and digital screen consumption. Analogue play is flexible and unstructured, involving physical movement and interaction, tactile sensations, and sensory engagement. These characteristics allow physical and cognitive exploration, emotional and social experiences. On the other hand, digital screens are characterized by high visual and auditory sensory outputs with fixed stimulation and predetermined outcomes. They direct attention through specific pathways, restricting freedom for imagination and exploration, and do not involve physical movement, face-to-face interaction, and multisensory experiences like the natural world. Consequently, excessive screen use is negatively associated with various developmental outcomes such as motor functions, social interactions, attention span, language acquisition, and emotional regulation (Anjum et al., 2025). With continuous structural progression and technological advancement, children are physically and sensorily enclaved in a digital environment. This creates developmental concerns among contemporary families struggling to alleviate the detrimental outcomes.

## 4 The Three-Layered Framework and the SMART protocol

Operational instructions for caregivers remain a critical missing component within existing ecological frameworks of early childhood screen use. Existing ecological and family-centered frameworks recognize parents as influential but seldom provide actionable steps that successfully integrate micro-level parenting factors into real-world behavior change, positioning parenting factors as a theoretical foundation for future longitudinal research and intervention development (Morawska et al., 2023). This pattern also appears in other frameworks, such as the Screen Time Ecological Model (STEM), which maps the environment and links it to intervention strategies but still requires practitioner selection and clinical judgement rather than direct caregiver execution (Ragasa et al., 2024). Institutional guidelines face a similar barrier, specifying behavioral targets but often provide inadequate tools and methods for step-by-step, real-world implementation, leaving a gap between theoretical guidance and domestic practice (Anjum et al., 2025). These daily tool and time constraints may intensify the practical barriers to rule enforcement across different household income levels and parental employment structures (Mansor et al., 2021). Existing studies frequently analyze these family-level risk factors and socioeconomic determinants as isolated variables rather than as parts of a unified intervention strategy (Gomes et al., 2026). This structural fragmentation may help explain why lower-income households frequently maintain awareness of restrictive guidelines, yet find that this awareness struggles to translate into active parental co-participation due to constrained household contexts (Hasanen et al., 2021).

The proposed Three-Layered Framework, accompanied by the SMART protocol, is intended to address these limitations by extending descriptive frameworks such as the AAP 2026 socioecological framework and existing institutional guidelines into an operationalized protocol that specifies step-by-step execution by caregivers across different environmental and resource constraints. While caregiver-oriented operational guidance addressing parental technoference already exists (Yelizarova et al., 2025), the contemporary descriptive literature offers limited task-oriented guidance for structured execution pathways tailored to diverse domestic environments and resource limitations. The SMART protocol integrates each component into an interdependent system, designed to reinforce the execution of each part to maintain behavioral balance under different environmental contexts. Building on this interdependence, the framework repositions caregivers not as one environmental factor among many, but as the primary factor reshaping the child’s micro-level environment, while shifting the practitioner’s role towards teaching and scaffolding, to support independent parental execution across different socio-demographic settings. Under this structural framework, the persisting digital silos may be transformed into shared spaces that foster children’s developmental resilience, supporting a shift from isolated screen use toward more restorative interaction with the environment. Unlike existing frameworks that isolate environmental factors, intervention, and developmental outcomes as separate components (Morawska et al., 2023; Ragasa et al., 2024), this framework unifies them as three concentric layers within a single structure (Table 1).

**Table 1:** Comparative analysis of screen time guidelines, ecological frameworks, and the Three-Layered Framework with SMART protocol.

Criteria	WHO guidelines	IAP guidelines	CPS 4Ms	AAP 5Cs	Ecological/Family-centered frameworks (incl. AAP 2026)	Three-Layered Framework with SMART protocol	Contribution of the Three-Layered Framework
Main focus	Duration thresholds within a movement guideline	Duration limits, digital hygiene, safety rules	Behavioral principles for parental habit change	Conversational mnemonic across five behavioral domains	Maps predictor variables (Morawska, AAP 2026) or assigns practitioner-selected strategies (STEM)	Operationalized protocol which can be executed directly by the caregiver	Positions the caregiver and the child’s environment as the central unit of change, with specific steps to translate guideline goals into daily practice
Environmental/SES constraints	Not addressed. Explicitly out of scope	Not addressed. Uniform across households	Acknowledged only, then applied uniformly	Not addressed	Acknowledged only, tracked as a variable, not addressed	Yes, tailored materials by setting (urban/rural)	Names low-cost materials and activities adapted to space-, time-, and resource-constrained households
Caregiver-level recommendations	Enforces a duration limit	Designates screen-free zones/times	Sets a family media plan and screen-free times	Reflects on what screen time displaces and considers alternatives	Important variable (Morawska, AAP 2026) or executor of practitioner-assigned strategies (STEM)	Directly controls and restructures the child’s screens environment	Positions the caregiver as the direct agent of change, not a variable among several or a delegate of assigned strategies
Degree of operationalization	Not operationalized	Partial, rules usable, relational goals undefined	Partial, thresholds specific, methods undefined	Partial, targets named, alternatives undefined	Partial to not operationalized, depending on framework	Operationalized, Named activities and materials.	Names concrete materials and techniques over general categories, enabling replication without practitioner interpretation
Remaining implementation gaps	No caregiver-facing tool	No definition of interactive co-viewing	No alternatives, no accommodation for absent parent	No systematic dependence-reduction method; alternatives stay general	Fragmented, no independent parent execution pathway	Not yet tested with families; effectiveness, feasibility, and caregiver adherence remain unconfirmed	Execution pathway defined; remaining gap is field validation, not method absence

SMART is designed using plain, jargon-free language so that caregivers across varying levels of literacy and resources can follow directly.

To map the proposed three-layered structure, the outer layer captures the environment that sets the foundation for screen habits and physical play, depicting the unmediated realities that parents battle against to reach the target mediated goal of the environment. This environmental division dictates the consequences of how physical interactions and screens are used. The middle layer embodies the transition from the unmediated to mediated environment by providing the essential process for selecting the appropriate tools to implement based on the individual requirements of children, the socio-geographical space constraints, and the availability of resources. This layer also integrates traditional play and provides analogue game ideas, including play materials, as alternatives to screen time. The SMART protocol, first specified in the author’s patent application (Lalnunmawii and Gangadgaran, 2025), clearly operationalizes the second layer, comprising five core components which are Screen awareness, Meaningful alternatives, Active engagement, Responsive caregiving, and Traditional game integration. The inner layer depicts the developmental outcomes of children based on the content and context of screen use and their interactions with the immediate environment, leading to bidirectional developmental milestones. This shows how the form of engagement can result in either detrimental effects or positive outcomes, potentially influencing self-regulation and overall well-being as children transition into later stages of life (Etokabeka, 2025; Howard et al., 2021).

To initiate the transition using the SMART protocol, establishing screen awareness helps parents consider not only the surface-level content but also make a clear distinction between the thematic qualities and formal structural features. It provides practical guidance to evaluate a program’s visual rhythm and structural pacing by recognizing rapid editing cuts or sudden fluctuations in volume, which helps parents distinguish lower-stimulation media from overstimulating content. Exclusive child-appropriate content can still present risk if visual pacing and auditory fluctuations are not taken into account, potentially creating sensory overstimulation (Namazi and Sadeghi, 2024). The current guidelines advise parents to filter the quality of content through general themes such as age-appropriate, educational, commercial, and violent content, but they offer no operationalization for selection, overlooking important elements like the structural features of the content. Regardless of screen awareness of quality content, in practice, parents may still face difficulties in the selection process due to daily time constraints. Families with limited time or digital literacy can apply this check informally by briefly observing the first few minutes of content, while families with access to smart devices or subscription platforms may rely on built-in content filters calibrated to similar pacing principles.

Families also face challenges such as the lack of practical ideas for low-cost, alternative activities, creating difficulties for those with limited resources. Due to space constraints, parents struggle to ensure that their children’s physical activities are not displaced by screen time (Lang et al., 2024). The absence of practical recommendations to address these challenges creates a disparity between families with open space and resources and families living in constrained spaces or with limited resources. This can contribute to parental strain and frustration under congested living conditions where screen time alternatives are physically restricted. To provide meaningful alternatives to passive screen time within these limitations, families with compact living spaces should be equipped with practical ideas that include indoor sensory engagement, compact physical play, and domestic chores as games to circumvent architectural congestion. However, rural areas demand the creative use of available, everyday items. This framework operationalizes replacement through accessible materials like atta for play dough, empty jars for arranging and bowling, cut-out newspaper pictures for sorting, rice for sensory play, and natural components such as stones, leaves, and sticks for unstructured play.

Active engagement operationalizes parental co-participation by changing the focus from continuous supervision to flexible, intermittent verbal check-ins that address the domestic reality where screens are frequently used as a babysitting tool to balance work and domestic chores (Nikken, 2019). Techniques like co-viewing are suggested for active engagement during screen time, which requires physical presence. Alternative techniques to active screen time participation should be introduced to avoid passive viewing when parents are occupied. Active engagement during screen use can also be achieved through practical interactions, active verbal engagement, question prompting, and scaffolding to connect the content to the real world (Ewin et al., 2021; Howard et al., 2024). For example, a parent between household chores can actively prompt questions while the child is engaged with the screen, providing parents with practical ways to connect with the child. Caregivers with constrained schedules or rigid working hours can adapt this technique by delivering brief, targeted question prompts during transitional moments of their work or domestic routines, ensuring that active engagement does not depend entirely on a continuous physical presence throughout screen engagement.

Responsive caregiving redirects the caregiver-child relationship away from digital screen dependency by teaching parents to actively monitor and understand their child’s situational and emotional needs. Children turn to screens for comfort in the absence of caregiver availability (Gath et al., 2023). Caregivers can evaluate these underlying behaviors by identifying the Antecedents, Behavior, and Consequences, starting with what leads to a screen request, such as boredom, a lack of alternative activities, or a lack of playmates, so they can address the underlying cause and aim to reduce the likelihood of future requests. This also involves observing how the child responds when screen time ends, potentially helping caregivers to reduce conflict during this transition by preparing the child in advance or offering an immediate alternative rather than an abrupt removal of the device. Caregivers with constrained time can monitor these during brief, specific windows around screen transitions which require no financial cost or structural changes.

Traditional games revive physical, analogue play that requires little to no manufactured toys. For instance, five stones, Chinese jump rope, and seed-knocking games require only a small space, making them practical for constrained households, while run-and-catch, hide-and-seek, and freeze games need no materials but require more open space. This resourceful approach creates an opportunity for children to interact with their immediate environment while facilitating their development.

The SMART protocol utilizes distinct delivery methods designed to match different levels of digital literacy and healthcare access. For families with time constraints or residing in high-density urban environments, the protocol is introduced as psychoeducation delivered using infographic modules or short video guides explaining implementation. Families with consistent access to institutional health infrastructure receive the protocol with printed manuals and detailed, one-on-one sessions through healthcare practitioners offered during in-person visits. Utilizing these diverse, accessible pathways ensures that both the conceptual structure of the Three-Layered Framework and the practical steps of the SMART protocol can be systematically evaluated across distinct urban, rural, and socioeconomic contexts.

## 5 Discussion

To achieve the current guidelines’ goals of reducing screen time and protecting child development, recommendations should not only outline the targets but also provide clear steps on how it can be achieved. Stakeholders, pediatric care providers, and healthcare institutions should incorporate the socio-geographic context and resource distribution in future guideline recommendations to provide equitable accessibility and application for different families living in diverse socioeconomic settings. Institutions such as the WHO, AAP, CPS, and IAP have provided a necessary starting point by establishing that screen exposure in early childhood requires structured clinical attention. They have also played an important role in transitioning attention from duration of screen time toward the content and context of use. Singapore has followed similar guidelines, where health institutions provide personalized health plans and lifestyle prescriptions based on parental reports of habits, which has resulted in 70.2% of young children following the guidelines (Chen et al., 2019). An effective guideline must encompass realistic, practical implementation strategies over generalized recommendations to eliminate the struggle that families face in following the current recommendations. Healthcare providers should shift their perspective from treating screen time problems as solely behavioral issues to addressing the main determinants of such screen habits instead. This perspective can better enable practitioners to understand the specific constraints of families who struggle with excessive screen time in order to provide actionable solutions.

Enforcing screen time guidelines and conceptual recommendations without considering the ground realities can compromise the main purpose of facilitating healthy child development. These passive institutional recommendations should be operationalized through an actionable structure like the proposed Three-Layered framework with the SMART protocol that provides a clear connection to real-world settings with operational modalities. New or evolved guidelines will not succeed without navigating the impact of the surrounding environment and providing proper education for families and the requirements for healthcare practitioners to execute the steps to reach the guidelines’ targets. Stakeholders and policy makers should establish a requirement for healthcare practitioners to start from environmental modifications before jumping straight into parenting practices and behavioral controls to break the cycle of screen time concerns among families. The Three-Layered Framework and SMART protocol proposed in this paper remain conceptual and have not yet been empirically tested. It is recommended that future research should evaluate the framework using controlled trials across varied urban and rural socioeconomic settings before broader implementation.

## Data Availability

The original contributions presented in the study are included in the article/supplementary material, further inquiries can be directed to the corresponding author.
